# The Effectiveness of Metformin in Diabetes Prevention: A Systematic Review and Meta-Analysis

**DOI:** 10.7759/cureus.46108

**Published:** 2023-09-28

**Authors:** Dhara Patel, Ismat E Ayesha, Neetha R Monson, Nimra Klair, Utkarsh Patel, Ayushi Saxena, Pousette Hamid

**Affiliations:** 1 Internal Medicine, California Institute of Behavioral Neurosciences & Psychology, Fairfield, USA; 2 Medicine, California Institute of Behavioral Neurosciences & Psychology, Fairfield, USA; 3 Neurology, California Institute of Behavioral Neurosciences & Psychology, Fairfield, USA

**Keywords:** type 2 diabetes, insulin resistance, prevention of diabetes, pre-diabetes, metformin

## Abstract

Diabetes mellitus is a growing global health concern, and prevention strategies play a crucial role in reducing its burden. Metformin has been widely studied as a potential intervention for diabetes prevention, but its overall effectiveness and impact on various populations remain unclear. This study aims to provide a comprehensive synthesis of the available evidence on the effectiveness of metformin in diabetes prevention. A systematic search was conducted in PubMed, Scopus, ScienceDirect, and Google Scholar for articles published from inception to June 2023. The reference lists of the included studies were also searched to retrieve possible additional studies. Any quantitative data were analyzed using Review Manager 5.4. A *P*-value of 0.05 was adopted as the significance threshold. Our analysis included 17 studies with a total sample size of 30,474. Our meta-analysis included two key analyses. First, the meta-analysis evaluating the effects of metformin on prediabetes demonstrated a significant reduction in the risk of progressing to type 2 diabetes mellitus (T2DM). The pooled odds ratio (OR) was 0.65 (95% confidence interval [CI] 0.53-0.80), indicating a 35% lower odds of developing T2DM among individuals with prediabetes who received metformin interventions compared to control groups. Secondly, the meta-analysis assessing the efficacy of metformin interventions in preventing T2DM yielded a significant reduction in the risk of developing the disease. The pooled risk ratio was 0.58 (95% CI 0.44-0.77), indicating a 42% lower risk of developing T2DM in individuals receiving metformin interventions compared to those in non-metformin intervention groups. These findings provide strong evidence for the effectiveness of metformin in preventing the progression of prediabetes to T2DM and reducing the overall incidence of the disease. The review demonstrated that metformin is effective in reducing the risk of developing diabetes mellitus among individuals at risk for the disease. The findings highlight the potential of metformin as a valuable intervention for diabetes prevention, particularly in high-risk populations.

## Introduction and background

Diabetes mellitus is a long-term metabolic condition characterized by high blood glucose levels [1]. It is a worldwide public health issue, affecting an estimated 463 million persons globally in 2019. This number is expected to rise to 578 million by 2030 and 700 million by 2045 [2]. Type 2 diabetes mellitus (T2DM), in particular, accounts for the vast majority of diabetes cases and is strongly linked to lifestyle variables such as obesity, sedentary activity, and poor eating habits. The concept of pre-disease, or at least its terminology, is relatively recent. The awareness that the top limits of normal measurements of blood glucose may pose a health concern and may be a warning that a patient is going toward diabetes is referred to as pre-disease [1].

Prediabetes is defined as a state of intermediate glucose dysregulation between normal glucose tolerance and overt diabetes [3]. Individuals with prediabetes have higher-than-normal blood glucose levels but have not yet reached the diabetes diagnosis threshold [3,4]. Impaired fasting glucose (IFG) and impaired glucose tolerance (IGT) are terms used to describe prediabetes. In 2021, 464 million and 298 million adults aged between 20 and 79 years were estimated to have IGT and IFG, respectively [5]. This is expected to increase to 638 million for IGT cases and 414 million for IFG cases by 2045 [5]. Prediabetes has been associated with the development of certain pathologies. Wen et al. reported on an 11-year follow-up of 36,000 people in 2005 that those with IFG (fasting glucose levels between 6.1 and 6.9 mmol/L) had a significantly higher risk of mortality from cardiovascular diseases (CVDs) and diabetes than those with blood glucose levels below 6 mmol/L [6]. Unwin et al. determined in a careful study of the matter that IFG and IGT (glucose 7.8 and 11.1 mmol/L, two hours after consumption of a 75-g oral glucose load) were strongly linked with CVD [7]. IGT was found to be more significantly linked to CVD than IFG [7]. Importantly, prediabetes increases the chance of developing T2DM, CVD, and other problems [1,6-10]. Identifying effective therapies to prevent or delay the transition from prediabetes to diabetes is, therefore, critical.

Adopting a balanced diet, increased physical exercise, and obtaining and maintaining a healthy weight are all considered the cornerstones of diabetes prevention. Lifestyle therapies have been shown in large-scale clinical trials, such as the Diabetes Prevention Program (DPP), to reduce the risk of developing diabetes by 58% in those with prediabetes [11]. Nonetheless, despite the established benefits of lifestyle changes, their execution and long-term adherence remain difficult for many people [11]. Pharmacological therapies have been examined as a viable method for diabetes prevention. Metformin, an oral antidiabetic drug, has emerged as a promising alternative among the numerous treatments investigated. Metformin is a biguanide derivative that reduces hepatic glucose synthesis, improves insulin sensitivity, and improves peripheral glucose uptake [12-14].

Metformin has been tested in various clinical trials for diabetes prevention, providing vital evidence of its efficacy and safety. The landmark DPP in the United States tested the efficacy of metformin, lifestyle changes, and placebo in people with prediabetes [11]. Over a two-year and eight-month average follow-up period, metformin lowered the chance of getting diabetes by 31% compared to a placebo group. Furthermore, a 10-year follow-up trial called the DPP outcomes trial (DPPOS) investigated the long-term benefits of metformin and found a persistent reduction in the incidence of diabetes. Metformin lowered the risk of acquiring diabetes by 18% over 10 years [15]. The objective of this systematic review and meta-analysis is to offer complete evidence on metformin's efficacy, safety, and long-term results by only using randomized controlled trials (RCTs).

## Review

Methodology

This review followed Preferred Reporting Items for Systematic Reviews and Meta-Analysis (PRISMA) 2020 guidelines [16]. Three databases (PubMed, Scopus, and ScienceDirect) were searched for articles comprehensively. The search was performed from the inception of the database to the present date. There was no restriction on the publication language, and non-English articles were translated. The articles had to present information on the effectiveness of metformin in diabetes prevention. PubMed was the primary database considered to represent internationally indexed articles across the globe.

Search Strategy

The search strategy for PubMed featured a combination of free keyword searches and controlled the Medical Subject Headings (MeSH) terms. The keyword search featured an all-text analysis to broaden the sensitivity of the search strategy. The search strategies used for Scopus and ScienceDirect were slight modifications of PubMed’s strategy. Table 1 represents the selection of primary keywords considered for PubMed search.

**Table 1:** Search strings. No data range was used in any of the index databases.

Database	Search field	Search string
PubMed	Title, abstract	(("metformin" OR "glucophage") AND ("efficacy" OR "effectiveness" OR "effect" OR "outcome" OR "benefit") AND ("safety" OR "adverse events" OR "side effects") AND ("long-term" OR "longitudinal" OR "prolonged" OR "sustained") AND ("diabetes" OR "prediabetes" OR "glucose intolerance" OR "insulin resistance" OR "type 2 diabetes" OR "T2DM") AND ("prevention" OR "preventing" OR "delaying" OR "reducing"))
Scopus	All fields	(Metformin OR Glucophage) AND (efficacy OR effectiveness) AND (safety OR adverse effects OR side effects) AND (long-term OR durability OR follow-up) AND (diabetes prevention)
ScienceDirect	Research articles	(Metformin) AND (efficacy OR effectiveness) AND (safety OR adverse effects OR side effects) AND (long-term OR follow-up) AND (diabetes prevention)

In addition to the search conducted on the three databases, a direct search was done using the Google Scholar database. To allow the presentation of the most relevant results on the first pages, keywords representing our research objective (metformin, diabetes prevention, prediabetes, T2DM, glucose metabolism, insulin resistance, and glycemic control) were included in the search. The reference lists of the included studies were also searched for any relevant additional articles.

Study Selection and Eligibility Criteria

To ensure the inclusion of reliable and robust evidence, only RCTs were chosen. The selected studies specifically involved the administration of metformin intending to delay or prevent the onset of T2DM. The participants in these studies were individuals with IGT or IFG, either in the entire sample or a subset. A crucial requirement was that the development of diabetes was measured as an outcome. Furthermore, the studies included in the review had a minimum follow-up period of six months.

Studies that satisfied the following criteria were excluded, studies that did not directly address the research question or did not meet the inclusion criteria defined in the review, non-primary studies, and studies with inadequate follow-up.

Potentially eligible studies were individually screened using Zotero (Corporation for Digital Scholarship, Fairfax, VA). The selection featured a rigorous screening of titles, abstracts, and full texts. The full-text screening featured a focus on the presentation of any form of data on the effectiveness of metformin in diabetes prevention. After the selection of articles for inclusion, data were extracted into a predefined data descriptor table with fields such as author, year of publication, study design, patients' characteristics, treatment group, control group, and outcome of the interest.

Quality Assessment and Statistical Analysis

The methodological quality of the included studies was assessed using the risk of bias (RoB) using the Cochrane tool for RCTs. For each study, we assessed the overall RoB score and categorized it based on the number of criteria for high RoB met. If a study fulfilled more than two criteria for *some concerns* of bias, we assigned it a *high* rating. If a study met one to two criteria for high RoB, we assigned it a rating of *some concerns*. Finally, if a study did not meet any criteria for a high RoB, we assigned it a rating of *low RoB*.

We employed random-effects meta-analysis models to consider the variability among studies. In cases where trials reported zero events in one of the study arms, we applied a continuity correction of 0.5. Our analysis involved estimating the combined relative risk (RR) for diabetes achieved after active intervention of medication trials. Additionally, we calculated the measure of association (odds ratio) between the intervention and the outcomes. To explore the effects of interventions after treatment withdrawal, we estimated the aggregate RR for diabetes at the end of active intervention, as well as at the end of the washout period for medication trials or the end of the follow-up period for lifestyle modification (LSM) trials. We assessed heterogeneity across studies by calculating I2, where a value greater than 75% indicated significant heterogeneity. For quantitative data analysis, we utilized Review Manager 5.4 software. A *P*-value of 0.05 was adopted as the significance threshold.

Results

**Figure 1:**
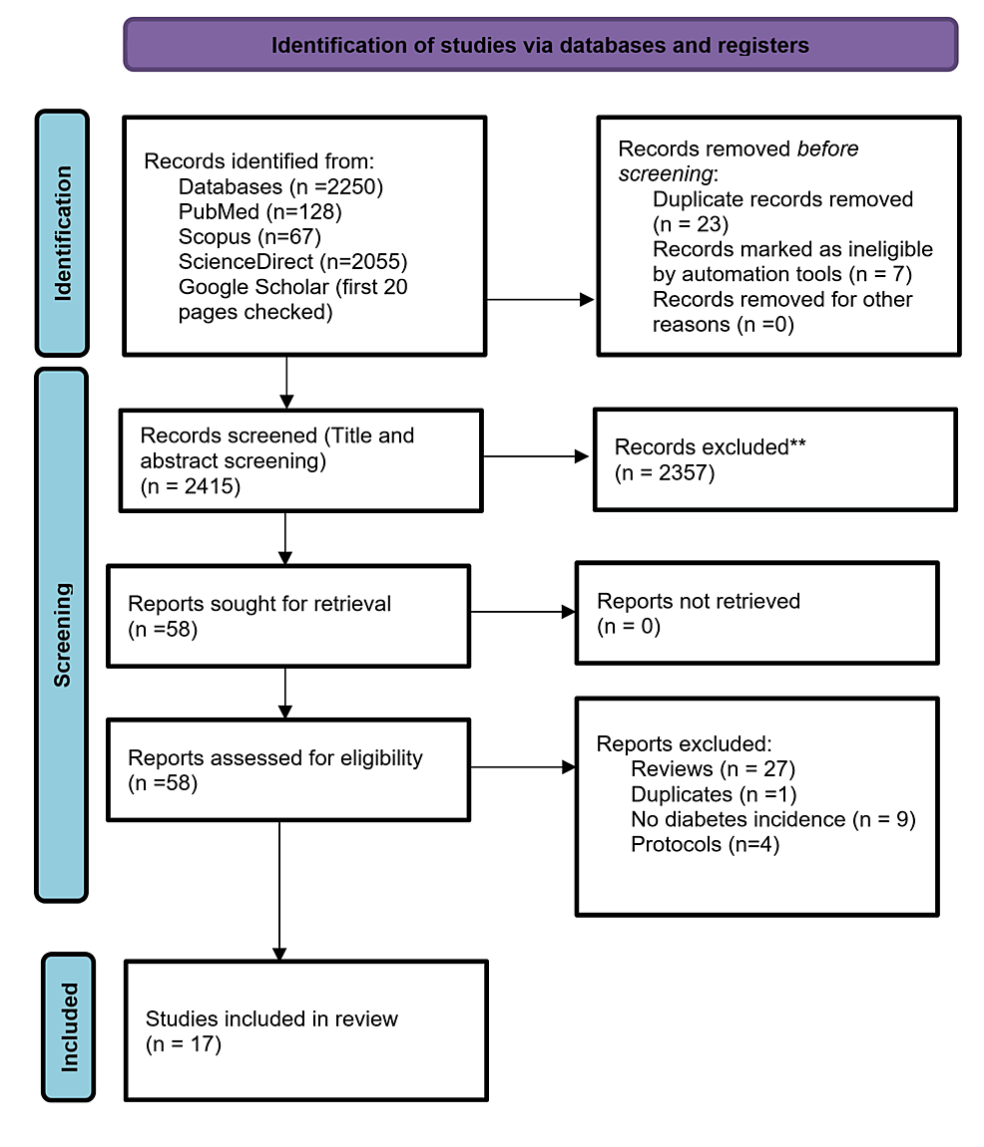
PRISMA flowchart. PRISMA, Preferred Reporting Items for Systematic Reviews and Meta-Analyses

Initially, a total of 2,250 articles were retrieved through the search process. After removing 23 duplicates, 2,357 articles were excluded based on the eligibility criteria during the title and abstract screening. The remaining 58 articles underwent a full-text review. Among them, 41 articles were excluded as they did not fully meet the eligibility criteria. It's important to note that some articles were excluded for multiple reasons. The specific reasons for exclusion are outlined in the PRISMA flowchart (Figure 1). Ultimately, only 17 articles met all the inclusion criteria and were included in the review paper. A visual representation of the study selection process is shown in Figure 1.

Study Characteristics

We included a total of 17 RCTs. The studies encompassed a sample size of 30,474 patients, with a mean age of 46.67 years. Among the participants, 14,628 (48.0%) were men, and the baseline body mass index averaged 30.8 (calculated as weight in kilograms divided by height in meters squared). The included articles involved participants from Asian, North American, and European regions. Among the included articles, five studies were not included in the meta-analysis for various reasons. Some studies did not report the number of participants who developed diabetes at the end of the study, while others did not provide the number of individuals at risk for diabetes at baseline in each arm (*n* = 1). On the other hand, 11 articles provided sufficient data for meta-analyses, and based on quality assessment, it was deemed appropriate to pool the data from these studies. The included studies had varying lengths of follow-up, with 13 studies ranging from 0.5 to 6.3 years. Notably, the US DPP studies reported a follow-up period of 10 years since randomization. Study characteristics and findings are summarized in Table 2.

**Table 2:** Study descriptor table. BID, twice daily; BMI, body mass index; FPG, fasting plasma glucose; IGT, impaired glucose tolerance; IFG, impaired fasting glucose; LSM, lifestyle modification; OGTT, oral glucose tolerance test; RCT, randomized controlled trial; TID, three times daily; DPP, Diabetes Prevention Program; ILS, intensive lifestyle; MET, metformin; IGR, impaired glucose regulation; GDM, gestational diabetes mellitus; IQR, interquartile range; HRQoL, health-related quality of life; *N*, total number of individuals or observations in the sample; RRR, relative risk reduction

Study	Study design	Patients	Treatment group	Control group	Outcome of interest
Knowler et al. [17]	A multicenter RCT with three arms: placebo group, MET group, and LSM group	Adults 25 years or older (mean age 51 years) with FPG 5.3-6.9 mmol/L and two-hour postprandial glucose of 7.8-11.0 mmol/L; 32% of patients were men; participants had an average BMI of 34.	Received 850 mg MET BID and standard lifestyle recommendation (*N* = 1,073)	Received placebo tablets and standard lifestyle recommendations (*N* = 1,082)	Development of diabetes after three years
Ramachandran et al. [18]	RCT with four groups: usual care, LSM or MET, LSM, and MET	10,839 men and women, aged 33-55 years, from a middle-class Asian Indian population with no major illnesses and no pre-existing diagnosis of diabetes, were screened from March 2001 to July 2002; IGT was diagnosed based on two consecutive OGTTs (FPG < 7 mmol/L; two-hour postprandial glucose 7.8-11.0 mmol/L)	Received 250 mg MET BID; *N* = 136 (133 available for follow-up)	Received usual care (standard health care advice); *N* = 129 (128 available for follow-up)	Development of diabetes after three years
Li et al. [19]	Double-blind, placebo-controlled RCT	29,938 subjects from Shougang Corporation in Beijing, China, were screened with OGTT in 1992. Of those, 1,165 had IGT and were rescreened in 1994. Those already taking MET or who had renal, hepatic, or ischemic heart disease were excluded. After rescreening, 90 still had IGT. Participants included men and women aged 30-60 years.	Received 250 mg MET TID and diabetes education (information on diet, exercise, and healthy lifestyle) every three months; *N* = 45 (33 included in the primary analysis)	Received placebo tablets identical in appearance to MET, provided by the MET manufacturer, and the same diabetes education as the MET group; *N* = 45 (37 included in the primary analysis)	Development of diabetes after 12 months
Knowler et al. (Diabetes Prevention Program Research Group) [15]	RCT	All active DPP participants were eligible for continued follow-up. 2,766 of 3,150 (88%) enrolled for a median additional follow-up of 5.7 years (IQR 5.5-5.8); 910 participants were from the lifestyle; BMI = 31.1 kg/m^2^ in men and 34.2 kg/m^2^ in women.	MET treatment was continued in the original MET group (850 mg BID as tolerated); with participants unmasked to assignment, and the original lifestyle intervention group was offered additional lifestyle support; *N* = 924.	DPP lifestyle participants were also offered two group classes each comprising four sessions every year to reinvigorate their self-management behaviors for weight loss; *N* = 932.	Diabetes incidence in the 10 years since DPP randomization was reduced by 34% (24–42) in the lifestyle group and 18% (7–28) in the MET group compared with placebo.
Diabetes Prevention Program Research Group [20]	RCT	2,776 (88%) of the surviving DPP cohort were followed in the DPP outcome study (DPPOS 2002-2013) and analyzed by intention-to-treat based on the original DPP assignment. During DPPOS, the lifestyle group was offered lifestyle reinforcement semi-annually and the MET group received unmasked MET. After the first 24 weeks, individual and group sessions were used to reinforce LSM behaviors. The MET and placebo treatment groups were double-masked but, for practical reasons, the lifestyle group was not (mean age = 51 years; BMI = 34 kg/m^2^).	DPP compared MET at 850 mg twice per day; *N* = 926	Individual behavioral lifestyle; *N* = 915 for a normal lifestyle and *N* = 935 for placebo.	During 15 years of average follow-up, lifestyle intervention and MET reduced diabetes incidence rates by 27% (*P *< 0.0001) and 18% (*P *= 0.001), respectively, compared with the placebo group, with a decline in group differences over time. At year 15, the cumulative incidences of diabetes were 55%, 56%, and 62%, respectively.
Weber et al. [21]	RCT	Participants (63.2% male; mean age, 44.4 [SD 9.3] years) had a mean BMI of 27.9 (SD 3.7) kg/m^2^, and 30.2% had IFG, 29.7% had IGT, and 40.1% had IFG + IGT. The mean follow-up time was 2.54 years (range 4–48 months).	The stepwise intervention included lifestyle classes plus MET when needed; *N* = 283.	Control arm participants received the study site’s standard of care for prediabetes: a single day with one-on-one visits with a physician, a dietitian, and a fitness trainer and one group class on diabetes prevention (e.g., following a low-fat diet rich in complex carbohydrates and fresh fruits and vegetables, increasing physical activity); *N* = 293.	34.9% of control and 25.7% of intervention participants developed diabetes (*P* = 0.014); the RRR was 32% (95% CI 7-50), and the number needed to treat to prevent one case of diabetes was 9.8.
Iqbal Hydrie et al. [22]	RCT	The 317 IGT subjects were randomized into three groups: the control group was given standard medical advice, the LSM group was given intensive LSM advice, and the LSM + drug group was given ILS advice and MET 500 mg BID; followed for 18 months in Pakistan.	Endurance exercises such as walking, jogging, and cycling were recommended to improve fitness. Reinforcing behavior modification was done by advising on a healthy diet and physical activity for each subject in consequent sessions; *N* = 95.	Were given general diet and exercise information at baseline and followed at subsequent visits, but no intensive individual-specific counseling was given to them: *N* = 108 as a control group and *N* = 114 for a normal lifestyle.	A total of 47 incident cases of diabetes were diagnosed (overall incidence was four cases per 1,000 person-months with an incidence of 8.6 cases in the control group, 2.5 cases in the LSM, and 2.3 cases in the LSM + drug groups).
Andreadis et al. [23]	RCT	Participants were assigned to one of two interventions: either standard lifestyle recommendations and pharmacologic treatment of risk factors or standard lifestyle recommendations and pharmacologic treatment of risk factors plus MET at a daily dose of 850 mg. The mean age of the subjects was 53.5 (±0.7) in the MET and 51.7 (±0.9) in the non-MET group (*P* = 0.154), and the percentages of males were 47.4% and 51.7%. The mean BMI was 32.8 kg/m^2^ in the MET group and 32.2 kg/m^2 ^in the non-MET group.	Pharmacologic treatment of risk factors plus MET at a daily dose of 850 mg. Participants attended visits at three-month intervals until the completion of one year; *N* = 175	Did not receive MET; *N* = 190	After the 12-month follow-up, T2DM was observed in 1.1% of the subjects who received MET and 8.1% of those who did not. The risk of T2DM was significantly lower in the MET group (risk difference = −7.0% with 95% CI from −12.7% to −1.4%, *P* = 0.012).
Diabetes Prevention Program Research Group [24]	RCT	The overall analysis consisted of 1,803 participants (893 MET and 910 placebos), 1,274 of whom participated in the washout and 529 of whom had already developed diabetes. The duration of time from the last medication dose to the OGTT (i.e., the washout period) averaged 11 days in both treatment groups.	The MET group received 850 mg BID; *N* = 893.	The control group received a placebo; *N* = 910.	The primary analysis of the DPP demonstrated that MET decreased the risk of diabetes by 31%. The washout study shows that 26% of this effect can be accounted for by a pharmacological effect of MET that did not persist when the drug was stopped. After the washout, the incidence of diabetes was still reduced by 25%.
Florez et al. [25]	RCT	The DPP was conducted in 27 centers in the United States, in 3,234 non-diabetic persons with elevated fasting and post-load plasma glucose, mean age 51 years, mean BMI 34 kg/m^2^; 68% women, and 45% members of minority groups. Mean follow-up of 3.2 years	ILS program with the goals of at least 7% weight loss and 150 minutes of physical activity per week, MET 850 mg BID; *N* = 1,043	Normal lifestyle, *N* = 1048	Participants who experienced weight gain had significant worsening on the same HRQoL-specific domains when compared to those who had treatment-related (ILS or MET) weight loss.
Lu et al. [26]	RCT	Adults in Beijing, China, were screened for IGR using the 75 g OGTT. Participants with IGR received lifestyle and health education; those who still had IGR after one year were randomly assigned to either a routine care group or an intensive integrated intervention group.	For the group randomized to receive intensive integrated intervention, those with isolated-IGT received acarbose (50 mg TID) and those with isolated-IFG or IFG/IGT received MET (0.25 g TID); *N* = 95.	Control targets were FPG <6.1 mmol/L, two-hour postprandial plasma glucose (2hPG) <8.0 mmol/L, blood pressure <130/80 mmHg, total cholesterol (TC) <4.5 mmol/L, low-density lipoprotein cholesterol (LDL-c) <2.5 mmol/L, triglyceride (TG) <1.5 mmol/L, and BMI <25 kg/m^2^ or weight loss of approximately 5%-10%; *N* = 86.	Intensive integrated intervention may significantly decrease the conversion rate of IGR to T2DM and increase the conversion ratio to normal glucose tolerance.
O’Brien et al. [27]	RCT	Participants were randomly assigned to receive one of three interventions: MET 850 mg or placebo BID, or an ILS intervention. The mean participant follow-up was 2.8 years. Overall, half of the participants were aged 45-59 years, approximately two-thirds were women, and 39% were members of minority groups; *N* = 2,910	Received MET 850 mg BID; *N* = 983.	Placebo BID or an ILS intervention. Placebo (*N* = 967) and lifestyle intervention (*N* = 960)	47% of participants had completed college and 53% had not. Compared to placebo, lifestyle participants who had completed college demonstrated a 68% reduction in diabetes incidence (95% CI = 56-77), whereas those with less education experienced a 47% risk reduction (95% CI = 29-61). For MET participants, college graduates experienced a 49% RRR (95% CI = 33-62), compared to 23% (95% CI = 1-41) among those with lower educational attainment.
Orchard et al. [28]	RCT	Participants had IGT (World Health Organization criteria plus FPG level >=5.3 mmol/L [>=95 mg/dL) and were followed for a mean of 3.2 years after random assignment to ILS intervention, MET therapy, or placebo. Carried out at research- and community-based centers.	MET, 850 mg BID; *N* = 1,073	ILS intervention (*N* = 1,079) and placebo group (*N* = 1,082)	The metabolic syndrome affected approximately half of the participants in the DPP at baseline. Both lifestyle intervention and MET therapy reduced the development of the syndrome in the remaining participants.
Ratner et al. [29]	RCT	3,234 participants with IGT were identified, qualified as having IGT by a two-hour OGTT, and randomized to three different treatment groups (placebo, MET, and ILS) in 27 clinical centers throughout the United States. Of the 350 women with a history of GDM, 122 were assigned to placebo, 111 to MET, and 117 to ILS, whereas among the 1,416 women without a history of GDM, 487 were assigned to placebo, 464 to MET, and 465 to ILS.	Subjects were randomized to either standard lifestyle or placebo or MET therapy or an ILS intervention.	Standard lifestyle and placebo	Women with a history of GDM randomized to placebo had a crude incidence rate of diabetes 71% higher than that of women without such a history. Among women reporting a history of GDM, both ILS and MET therapy reduced the incidence of diabetes by approximately 50% compared with the placebo group, whereas this reduction was 49% and 14%, respectively in parous women without GDM.
Sussman et al. [30]	RCT	3,081 participants with impaired glucose metabolism at baseline, 655 (21%) progressed to diabetes over a median 2.8 years follow-up. Intervention groups received MET or an LSM program. All participants had a BMI of 24 kg/m^2 ^or higher (22 kg/m^2^ or higher in Asians) and an FPG concentration of 95 to 125 mg/dL (IFG) and 140 to 199 mg/dL two hours after a 75 g oral glucose load (IGT).	Standard lifestyle recommendations plus 850 mg of MET BID (*N* = 1,027)	Standard lifestyle recommendations plus placebo BID (*N* =1,030)	The benefit of MET, however, was seen almost entirely in patients in the top quarter of the risk of diabetes. No benefit was seen in the lowest-risk quarter. Participants in the highest-risk quarter averaged a 21.4% three-year absolute risk reduction (number needed to treat 4.6).
Zinman et al. [31]	RCT	RCT undertaken in clinics in Canadian centers, 207 patients with IGT were randomly assigned to receive a combination of rosiglitazone (2 mg) and MET (500 mg) BID or a matching placebo for a median of 3.9 years (IQR 3.0-4.6); *N *= 207 with IGT; BMI = 31.7 kg/m^2^.	Treatment received MET 500 mg and rosiglitazone 2 mg BID; *N* = 103.	The control group received a placebo; *N* = 104.	Incident diabetes occurred in significantly fewer individuals in the active treatment group (*N *= 14, 14%) than in the placebo group (*N *= 41, 39%; *P *< 0.0001). The RRR was 66% (95% CI 41-80) and the absolute risk reduction was 26% (95% CI 14-37), yielding a number needed to treat of four (2.70-7.14). 70 (80%) patients in the treatment group regressed to normal glucose tolerance compared with 52 (53%) in the placebo group (*P *= 0.0002).
Diabetes Prevention Program Research Group [32]	Randomized double-blind clinical trial	3,234 participants from 27 clinics in the United States were enrolled in the DPP; the 2,155 randomly assigned to the MET (1,073) or placebo (1,082) arms were included in this analysis. Participants were ≥25 years of age, had a BMI ≥24 kg/m^2^ (≥22 kg/m^2^ in Asian Americans), elevated fasting glucose (95-125 mg/dL), and IGT (140-199 mg/dL) two hours after a 75-g oral glucose load. Followed by a seven- to eight-year open-label extension and analysis of adverse events, tolerability, and the effect of adherence on change in weight and waist circumference.	MET or matching placebo was initiated at 850 mg once per day and increased by one month to 850 mg BID unless gastrointestinal symptoms warranted a longer titration period; *N* = 1,073	Normal lifestyle; *N* = 1,082	Throughout the unblinded follow-up, weight loss remained significantly greater in the MET group than in the placebo group (2.0% vs. 0.2%, *P* < 0.001), and this was related to the degree of continuing MET adherence (*P* < 0.001).

Results of Included Studies

In the study conducted by Li et al., a total of 90 participants were enrolled, with 45 assigned to each group. However, in their primary analysis, the authors excluded patients from both groups if they did not adhere to the treatment (metformin or placebo), were lost to follow-up, or experienced gastrointestinal side effects [19]. Consequently, only 70 patients were included in the primary analysis: 33 in the metformin group and 37 in the placebo group. Although the authors performed an intention-to-treat analysis, they still excluded five participants (three from the metformin group and two from the placebo group) who were lost to follow-up. The authors provided follow-up outcomes for those excluded due to non-compliance and side effects, but not for those lost to follow-up. In our report, we focused on the primary analysis of the 70 participants as reported by Li et al. [19]. However, we also conducted an intention-to-treat analysis, which included the five participants lost to follow-up.

In the study conducted by Ramachandran et al., a total of 531 participants were enrolled and randomly assigned to four different groups [18]. For our comparison, we focused on the metformin-only group and the usual care (control) group. Notably, the control group did not utilize a placebo, making it impossible to blind the patients to their respective treatments. Out of the 129 participants enrolled in the control group and 136 in the metformin group, only 128 and 133 participants, respectively, were available for follow-up and subsequent analysis.

The study conducted by Knowler et al., as part of the DPP Research Group, enrolled a total of 3,234 individuals who were randomly assigned to one of three groups [17]. In our analysis, we specifically compared the placebo-controlled group, consisting of 1,082 participants, with the metformin group, which comprised 1,073 participants. This study was notable for its well-executed design, comprehensive reporting, and notably larger sample size compared to other studies. The authors conducted an intention-to-treat analysis, which involved including all participants enrolled in the study when assessing the outcomes. Unfortunately, the authors did not provide the actual number of participants lost to follow-up in each group. However, they did report that 99.6% of the full study population was alive at the end of the follow-up period. Assuming this percentage was equally distributed across both groups, we used it to estimate the number of individuals lost to follow-up in each group.

**Figure 2:**
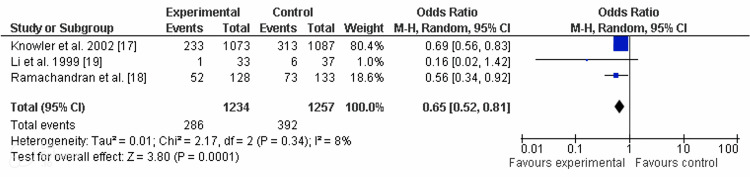
Forest plot. CI, confidence interval

**Figure 3:**
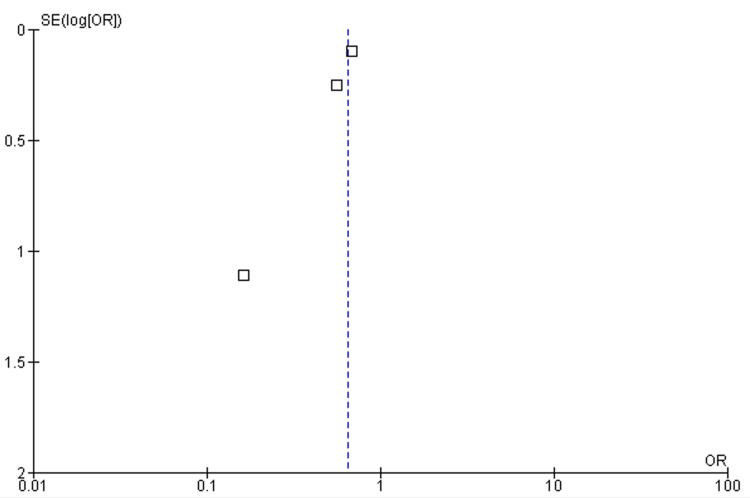
Funnel plot. OR, odds ratio; SE, standard error

One meta-analysis was performed. Figures 2-3 show the results of the meta-analysis that includes the findings from the three studies as the authors reported them.

Andreadis et al. conducted an RCT to examine the impact of including metformin in the treatment of overweight and obese individuals [23]. The study aimed to determine whether this addition would result in a further decrease in the occurrence of T2DM, prediabetes, metabolic syndrome (MetS), and improvements in risk factors for CVD. The findings of the study indicated that metformin supplementation reduces the likelihood of developing T2DM in overweight and obese individuals without diabetes, while also improving the MetS condition by enhancing the profile of CVD risk factors. Florez et al. conducted a study to evaluate the impact of interventions, specifically the use of metformin at a dosage of 850 mg twice daily, on changes in health-related quality of life (HRQoL) to reduce the risk of diabetes [25]. The researchers concluded that these interventions led to a beneficial decrease in diabetes occurrence. Iqbal Hydrie et al. conducted a study that highlighted the significant role of lifestyle intervention in preventing diabetes among individuals with IGT in a specific region [22]. The study found that the addition of medication did not yield any notable improvements. As a result, the researchers recommended the incorporation of lifestyle advice and follow-up as essential components of primary healthcare to address this issue effectively.

O'Brien et al. conducted a study to compare the effectiveness of intensive lifestyle intervention and metformin in individuals participating in the DPP, considering their level of education [27]. The researchers concluded that both intensive lifestyle intervention and metformin demonstrated higher efficacy among individuals with a higher level of education. Orchard et al. conducted a study to assess the prevalence of MetS at the start of the DPP and to examine how intensive lifestyle intervention and metformin therapy influenced the occurrence and resolution of the syndrome [28]. The study concluded that both the lifestyle intervention and metformin therapy contributed to a reduction in the development of MetS among the remaining participants. Ratner et al. conducted a study aiming to identify individuals who had IGT and intervene to prevent or delay the onset of diabetes [29]. The study findings indicated that both intensive lifestyle intervention and metformin were highly successful in postponing or preventing diabetes in women with IGT and a history of gestational diabetes mellitus (GDM). Sussman et al. conducted a study to investigate whether certain participants in the DPP would derive greater or lesser benefits from either metformin or a structured LSM program [30]. The study findings revealed that the benefit of metformin was predominantly observed in patients belonging to the highest quarter of diabetes risk. Conversely, no significant benefits were observed in the lowest-risk quarter. Participants in the highest-risk quarter experienced an average absolute risk reduction of 21.4% over three years, with a number needed to treat of 4.6.

Weber et al. conducted a study to evaluate the efficacy of expert guidelines for preventing diabetes, specifically focusing on lifestyle intervention and the addition of metformin as needed for individuals with prediabetes [21]. The study concluded that a majority of the participants required the inclusion of metformin in addition to lifestyle intervention. Zinman et al. conducted a study to examine the impact of low-dose combination therapy on the development of T2DM [31]. The study findings indicated that low-dose combination therapy involving rosiglitazone and metformin proved to be highly effective in preventing the onset of T2DM in patients with IGT. Additionally, the study noted minimal influence on the clinically significant adverse events associated with these two drugs.

The DPP research group conducted an RCT study in 2003 [24]. The study findings revealed that the primary analysis of the DPP demonstrated a 31% reduction in the risk of diabetes with the use of metformin. Another study by the DPP research group conducted in 2012 reported that the use of metformin for diabetes prevention is both safe and well-tolerated [32]. They also noted that weight loss is associated with adherence to metformin and remains consistent over a treatment period of at least 10 years. The DPP research group conducted a study in 2009, which revealed that over 10 years since the randomization of the DPP, the incidence of diabetes was reduced by 34% (with a range of 24%-42%) in the lifestyle intervention group and by 18% (with a range of 7%-28%) in the metformin group when compared to the placebo group [15]. In 2015, the DPP research group conducted a study to examine the lasting effects of lifestyle intervention and metformin in preventing diabetes, as initially demonstrated in the three-year DPP [20]. The study also aimed to determine if these interventions had a positive impact on diabetes-related microvascular complications. The findings of the study indicated that both lifestyle intervention and metformin significantly reduced the development of diabetes over 15 years.

Efficacy of Medication Interventions

The results of the meta-analysis indicate that the use of metformin medication interventions is associated with a significant reduction in the risk of developing diabetes. The risk ratio of 0.58 suggests that individuals receiving metformin had a 42% lower risk of developing diabetes compared to those in the control group. This finding highlights the efficacy of metformin as a preventive measure for diabetes. The 95% confidence interval (CI) of 0.44 to 0.77 indicates a high level of confidence that the true risk ratio falls within this range. These results suggest that metformin shows promise in reducing the risk of diabetes and could be considered an effective intervention in preventing the onset of the disease.

**Figure 4:**
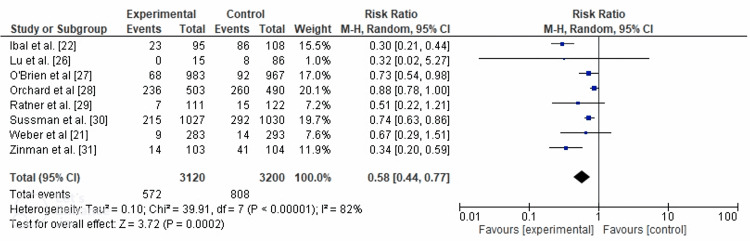
Forest plot for efficacy of medication intervention. CI, confidence interval

**Figure 5:**
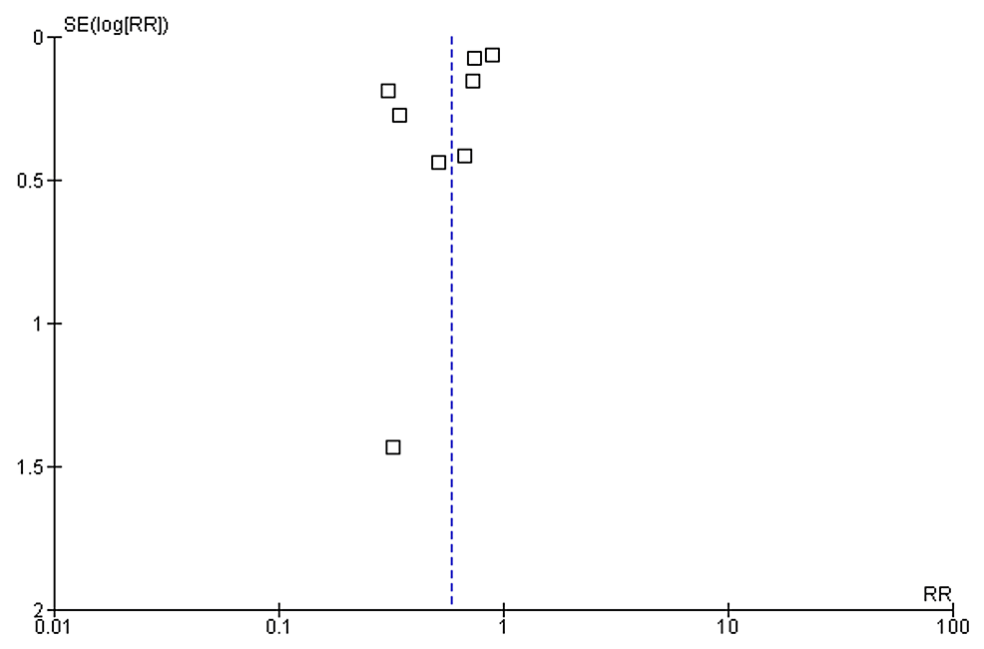
Funnel plot for the efficacy of medication intervention. RR, relative risk; SE, standard error

In the analysis, the heterogeneity statistic, represented by *I*², was calculated to be 82%. This meant that approximately 82% of the total variation in the study results could be attributed to factors other than random chance. In simpler terms, there is a substantial amount of diversity among the studies included in the meta-analysis. Additionally, the *P*-value associated with the heterogeneity test was 0.0002. The *P*-value indicated the statistical significance of the observed heterogeneity. In this case, the low *P*-value suggested that the observed heterogeneity is unlikely to have occurred by chance alone. Therefore, the differences among the study results are considered statistically significant. Forest and funnel plots for the efficacy of medication interventions are shown in Figures 4-5, respectively.

RoB in Included Studies

**Figure 6:**
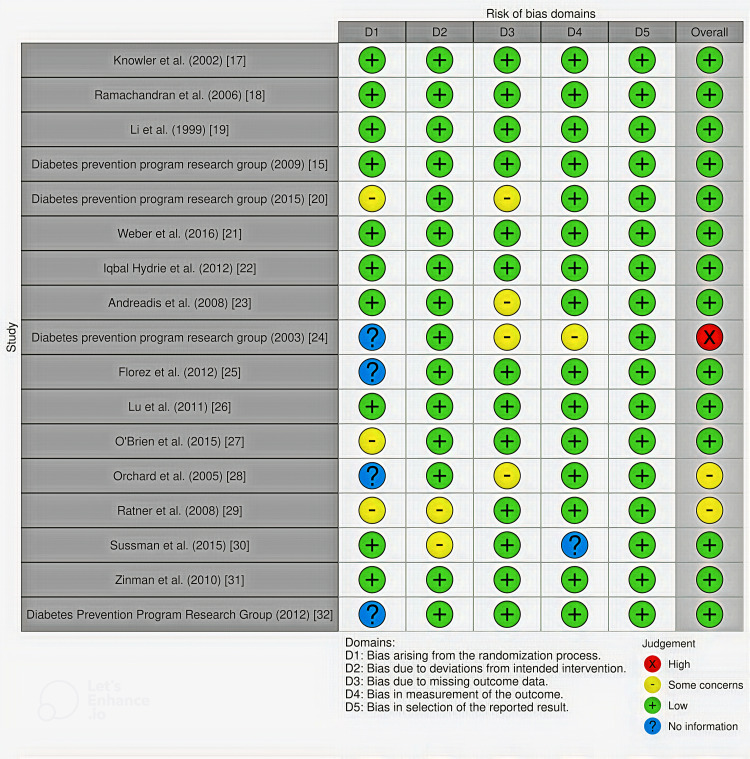
Traffic light plot.

**Figure 7:**
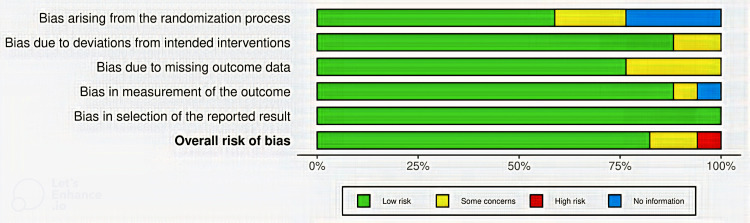
Summary plot.

The majority of the studies were determined to have a low RoB due to deviations from the intended intervention, bias in outcome measurement, and bias in the selection of reported results. On the other hand, several studies were identified to have *some concerns* regarding the RoB related to the randomization process. The traffic light plot and summary plot were generated as shown in Figures 6-7, respectively.

Discussion

The prevention of diabetes is of paramount importance in addressing the global burden of this chronic disease. Metformin, a widely prescribed oral medication for the treatment of T2DM, has gained significant attention for its potential role in diabetes prevention. In this systematic review and meta-analysis, we sought to evaluate the effectiveness of metformin in preventing the onset of diabetes in individuals at high risk. By synthesizing the available evidence from RCTs, our study provides valuable insights into the efficacy of metformin as a preventive intervention.

Our systematic review and meta-analysis encompassed a range of studies evaluating the effectiveness of metformin in diabetes prevention. The study conducted by Li et al. demonstrated that metformin reduced the risk of developing diabetes, as evidenced by the primary analysis of 70 participants [19]. Similarly, Ramachandran et al. found that metformin, compared to usual care, showed a potential for diabetes prevention, although the availability of participants for follow-up analysis was slightly limited [18]. Of particular note was the study conducted by Knowler et al., which encompassed a larger sample size and employed a robust design [17]. Their findings indicated a reduction in diabetes incidence by 18% to 34% in the metformin and lifestyle intervention groups, respectively, compared to the placebo group over 10 years. These results provide compelling evidence for the effectiveness of metformin in preventing diabetes in high-risk individuals. Furthermore, our analysis revealed consistent findings across multiple studies. Andreadis et al. reported that metformin supplementation significantly reduced the likelihood of developing T2DM in overweight and obese individuals without diabetes while also improving the profile of CVD risk factors [23]. Florez et al. highlighted the beneficial impact of interventions, including metformin, on reducing the occurrence of diabetes [25]. Conversely, Iqbal Hydrie et al. emphasized the significance of lifestyle intervention in preventing diabetes among individuals with IGT, suggesting that medication alone did not yield notable improvements [22]. In line with these findings, studies conducted by the DPP research group consistently demonstrated the effectiveness and long-term benefits of metformin in diabetes prevention. Their research showed a 31% reduction in diabetes risk with metformin use, as well as the safety and tolerability of metformin over an extended treatment period. Moreover, the group's studies showcased the lasting effects of lifestyle intervention and metformin, leading to significant reductions in diabetes development over 15 years, as well as potential positive impacts on diabetes-related microvascular complications.

Our meta-analysis revealed compelling evidence supporting the effectiveness of metformin in reducing the risk of developing diabetes. The analysis demonstrated a significant reduction in the risk of diabetes among individuals receiving metformin medication interventions, as indicated by the risk ratio of 0.58. This finding suggests that individuals receiving metformin had a 42% lower risk of developing diabetes compared to those in the control group. Collectively, these findings from various studies provide robust evidence supporting the effectiveness of metformin in preventing the onset of diabetes, particularly in high-risk individuals. The results highlight the potential of metformin as a valuable preventive intervention in the global efforts to combat the growing burden of diabetes.

Our findings are supported by several other studies investigating the effectiveness of metformin in diabetes prevention. Maruthur et al. conducted a systematic review and meta-analysis, which showed that metformin, was effective in reducing the incidence of diabetes in individuals with IGT, MetS, and polycystic ovary syndrome [33]. These findings support the broad applicability of metformin in various high-risk populations. Moreover, the Diabetes Prevention Program Outcomes Study (DPPOS) conducted by the DPP research group in 2009 demonstrated the long-term efficacy of metformin in reducing the risk of diabetes, with the benefits persisting over 10 years. Gebrie et al. conducted a network meta-analysis comparing various interventions and found that metformin was among the most effective interventions in reducing the incidence of diabetes [34]. This aligns with our results, indicating the efficacy of metformin in preventing T2D. Furthermore, Abbasi et al. conducted research focusing on the effects of metformin on CVDs in patients with diabetes [35]. Their analysis revealed that metformin use was associated with a reduced risk of CVD events and mortality in diabetic patients. Although our focus is on diabetes prevention, these findings suggest that metformin may provide additional benefits by reducing CVD risk factors in individuals with diabetes. Another systematic review and meta-analysis by Vella et al. analyzed the effects of metformin in preventing diabetes in individuals at high risk [36]. Their findings supported the notion that metformin significantly reduced the risk of diabetes compared to placebo or no treatment. This concurs with our results, reinforcing the role of metformin as an effective intervention for diabetes prevention. Furthermore, a study by Rena et al. explored the mechanisms of action of metformin and its role in preventing diabetes [37]. Their findings indicated that metformin improved insulin sensitivity, reduced hepatic glucose production, and decreased intestinal glucose absorption, all of which contribute to its preventive effects on diabetes. These mechanisms align with our findings; supporting the notion that metformin exerts its beneficial effects through multiple pathways.

This review has several limitations that should be considered. First, heterogeneity among the included studies, such as variations in participant characteristics, interventions, and outcome measures, may limit the ability to conduct a pooled analysis and draw definitive conclusions across all studies. However, we addressed these limitations by conducting a comprehensive search strategy across multiple databases and implementing strict inclusion criteria to minimize the risk of missing relevant studies and ensure the selection of high-quality research. Furthermore, our review included a meta-analysis, allowing for a quantitative synthesis of the data, and enhancing the statistical power of our analysis. Overall, the comprehensive search strategy, rigorous study selection, and meta-analysis were key strengths of our review, providing a solid foundation for the findings and contributing to the reliability and validity of our conclusions.

## Conclusions

This systematic review and meta-analysis provides strong evidence supporting the effectiveness of metformin in the prevention of diabetes mellitus. The findings from our analysis, along with the alignment with other relevant studies, consistently demonstrate that metformin supplementation reduces the likelihood of developing T2DM, particularly in overweight and obese individuals without diabetes. The combination of metformin and lifestyle intervention shows synergistic effects, emphasizing the importance of comprehensive approaches in diabetes prevention strategies. The consistent evidence from our review supports the consideration of metformin as a valuable intervention in diabetes prevention efforts, especially in high-risk populations. Further research should continue to explore the specific subgroups and factors that may influence the effectiveness of metformin, as well as long-term outcomes and potential adverse effects. Ultimately, the findings from our review contribute to the growing body of evidence, highlighting the important role of metformin in reducing the burden of T2DM and informing healthcare practitioners and policymakers in making informed decisions regarding diabetes prevention strategies.
